# The Incidence and Risk Factors for Persistent Acute Kidney Injury Following Total Cavopulmonary Connection Surgery: A Single-Center Retrospective Analysis of 465 Children

**DOI:** 10.3389/fped.2021.566195

**Published:** 2021-07-07

**Authors:** Yuan Jia, Qipeng Luo, Zhanhao Su, Chao Xiong, Hongbai Wang, Yinan Li, Xie Wu, Su Yuan, Fuxia Yan

**Affiliations:** ^1^Department of Anesthesiology, National Center of Cardiovascular Diseases, Fuwai Hospital, Chinese Academy of Medical Sciences and Peking Union Medical College, Beijing, China; ^2^Department of Pain Medicine, Peking University Third Hospital, Beijing, China; ^3^Center for Pediatric Cardiac Surgery, National Center of Cardiovascular Diseases, Fuwai Hospital, Chinese Academy of Medical Sciences and Peking Union Medical College, Beijing, China

**Keywords:** persistent acute kidney injury, pediatric patients, congenital heart disease, total cavopulmonary connection, renal perfusion pressure, risk factors

## Abstract

**Background:** Acute kidney injury (AKI) after cardiac surgery contributes to adverse outcomes. We aimed to assess the incidence and identify the predictors for persistent AKI after total cavopulmonary connection (TCPC) surgery.

**Methods:** A retrospective study, including 465 children undergoing TCPC surgery from 2010 to 2019, was conducted. We used pRIFLE criteria to define AKI and defined persistent AKI as AKIs occurring between post-operative day1 (POD1) and POD3 and sustaining at least on POD7. Univariate and multivariate logistic regressions were applied to analyze the predictors for persistent AKI.

**Results:** A total of 35.3% patients developed AKI between POD1 to POD3 and 15.5% patents had persistent AKI after TCPC. Patients with persistent AKI had prolonged mechanical ventilation and ICU stay, and had higher rates of renal replacement treatment and reintubation, which was associated with higher hospitalization costs and in-hospital mortality. The independent predictors for persistent AKI were peripheral oxygen saturation (SpO2) upon admission, intraoperative fluid balance, POD0 maximal lactic acid, renal perfusion pressure (RPP), POD0 estimated glomerular filtration rate and POD0 total bilirubin. The areas under receiver operating characteristic curve (AUC) in the total cohort and the subgroup undergoing TCPC surgery after 2017 were 0.75 (95% CI, 0.66–0.82) and 0.87 (95% CI, 0.77–0.97), respectively. The acceptable AUCs (nearly 0.7) were achieved in other 5 subgroups and good calibration ability (*p* ≥ 0.05) were achieved in the total cohort and all six subgroups.

**Conclusions:** Persistent AKI after TCPC was common and strongly associated with poorer in-hospital outcomes in Chinese pediatric patients. Six perioperative variables, including SpO2, intraoperative fluid balance, POD0 maximal lactic acid, RPP, POD0 moderate-to-severe kidney injury and POD0 total bilirubin, were identified as independent predictors for persistent AKI. Our findings may help to perform an early risk stratification for these vulnerable patients and improve their outcomes.

## Introduction

Acute kidney injury (AKI) after cardiac surgery is strongly linked to adverse outcomes, including the development of chronic kidney disease, and higher short and long-term mortality (1–5). Pediatric patients are susceptible to AKI after cardiac surgery with wide range of incidences (15–52%) (2, 6, 7), especially complex congenital heart surgeries, like the total cavopulmonary connection (TCPC), a common palliative procedure for functionally single ventricle patients. The circulation in patients after TCPC is characterized by relatively increased central venous pressure and susceptibility to low cardiac output, which may reduce renal perfusion pressure (RPP) and increases the risk of kidney injury (8).

In previous studies, the identified predictors for AKI in post-operative day 1–3 (POD1-3) after lateral tunnel or extracardiac TCPC included cardiopulmonary bypass (CPB) time, pre-operative pulmonary resistance, RPP, higher vasoactive inotropic use and atrioventricular valve regurgitation (9, 10). However, these studies are limited by failing to assess other important variables that may be associated with higher risk of AKI, such as some immediate post-operative biochemical variables which reflect the extent of hepatic injuries. Furthermore, the predictors for persistent AKI (AKIs sustaining at least on POD7) after TCPC remain unclear, and the predictors for AKI after TCPC surgery in a population of different characteristics in China, including relatively old operation age, more patients with single stage TCPC and different predominant diagnosis, are also unknown.

To address these current knowledge gaps, we aim to assess the incidence of persistent AKI and identify predictors for persistent AKI in a Chinese pediatric population undergoing TCPC surgery.

## Materials and Methods

### Study Population

We retrospectively included patients under 18 years old who underwent TCPC surgery in our center. Patients were excluded if they died within 7 days of surgery because of the missing value of creatinine after post-operative day 7 in these patients, if they had extracorporeal membrane oxygenation within 7 days of surgery because the creatinine levels were diluted in patients with extracorporeal membrane oxygenation, or if they had missing values of post-operative creatinine level. The study was approved by the Research Ethics Board of Fuwai Hospital and all data were retrieved from electronic medical records. Informed consents were waived because this study was a retrospective study.

### Definition of Persistent Acute Kidney Injury

We used the Schwartz model formula to calculate the estimated creatinine clearance (eCCI) (11) and the presence of AKI was determined by the pediatric-modified risk, injury, failure, loss and end-stage renal disease (pRIFLE) criteria, which including the following AKI: risk(AKI-R), injury(AKI-I), and failure(AKI-F) (12). The pRIFLE criteria categorizes three levels of AKI based on the percentage decline of eCCI: AKI-R (decline of 25–50%), AKI-I (decline of 50–75%) and AKI-F (decline > 75%). Patients, who developed AKI between POD1 and POD3 and still diagnosed with AKI between POD7 and POD9, were defined as having persistent AKI (13). The diagnosis of AKI between POD1 to POD3 was based on the maximal creatinine level collected between POD1 and POD3. The serum creatinine (SCr) used for the definition of persistent AKI were measured between POD7 and POD9.

### Data Collection and Variable Definition

All candidate variables were included on the basis of clinical relevance, literature review and data availability in our hospital. The pre-operative variables were recorded: age at operation, weight, gender, body surface area, peripheral oxygen saturation (SpO2) on room air before surgery, pre-operative pulmonary artery pressure, prior Glenn, prior B-T shunt, diagnosis of heterotaxy syndrome, original anatomy and ventricle morphology. The following pre-operative and POD0 blood laboratory biochemical variables were considered: counts of white blood cell, neutrophil, lymphocyte, monocyte and platelet, levels of hemoglobin, prothrombin time, total protein, albumin, alanine aminotransferase, aspartate aminotransferase, alkaline phosphatase, total bilirubin, SCr, isoenzyme of creatine kinase-MB, lactate dehydrogenase, and high-sensitivity C-reactive protein. The following pre-operative echocardiography parameters were collected: systemic ventricle ejection fraction, systemic ventricle end-diastolic diameter z-score, atrioventricular valve regurgitation and Nakata index from cardiovascular CT or catheterization. Preoperative pulmonary artery pressure was mostly achieved from cardiac catheterization test, and for some patients without cardiac catheterization test and with prior Glenn surgery, it was from internal jugular venous pressure before the surgery.

Intraoperative anesthesia and surgery variables included senior surgeons who performed an average of TCPC procedures over 10 cases per year, SpO2 inhaling 100% oxygen after intubation, use of milrinone, levosimendan, epinephrine, norepinephrine, sodium bicarbonate and vasopressin, transfusion of red blood cell, fresh frozen plasma and platelet, CPB time, aortic cross-clamping time, creation of fenestration, internal tunnel procedure, concomitant atrioventricular valve surgery, minimal temperature during CPB, maximum vasoactive-Inotropic score during surgery, SpO2 inhaling 50–60% oxygen after surgery, and intraoperative fluid balance. The following post-operative variables were also collected: POD0 maximal lactic acid, RPP when admission to ICU, POD0 fluid balance, and post-operative pulmonary artery pressure when admission to ICU. The pre- and post-operative variables were measured at the nearest moment before and after the surgery, respectively. POD0 was defined as the time period between post-operative admission into ICU and 8:00 A.M. in the next morning.

Fluid balance (mL/kg) was calculated: Fluid balance = [(amount of crystalloids + colloids + packed red cell + plasma + platelets + enteral nutrition)–(blood loss + urine output + gastrointestinal losses + drain losses + dialysis or ultrafiltrate)]/weight. Z-scores indexed to body surface area were applied when calculating left ventricular end-diastolic diameter z-score. Estimated glomerular filtration rate (eGFR) was computed: eGFR = 0.413^*^height (cm) / serum creatinine (SCr) level (mg/dL). Vasoactive-Inotropic score was calculated: dopamine (μg/kg/min) + dobutamine (μg/kg/min) + 10 ^*^ milrinone (μg/kg/min) + 50 ^*^ levosimendan (μg/kg/min) + 100 ^*^ epinephrine (μg/kg/min) + 100 ^*^ norepinephrine (μg/kg/min) + 10,000 ^*^ vasopressin (U/kg/min) (14). RPP was defined as mean arterial pressure minus central venous pressure (10). Renal replacement treatment included peritoneal dialysis and blood filtration.

### Management After TCPC Surgery

When patients were admitted to the ICU after TCPC surgery, blood tests were routinely performed at three time points in our hospital: POD0, POD1-POD3, POD7-POD9. Therefore, we determined the presence of persistent AKI based on the serum creatinine level during POD7 and POD9. The treatments including diuretics, anti-inflammation, renal replacement treatment were administered in patients with AKI if necessary and the treatments including intravenous treprostinil, oral administration of endothelin receptor antagonists (bosentan, macitentan), inhalation of nitric oxide, appropriate sedation, creation of fenestration, etc. for improving post-operative circulation and reducing pulmonary vascular resistance were administered if necessary. Dexamethasone (0.3 mL/kg) was routinely administered in almost all patients during operation or upon admission to ICU. The decision of chest tube removal was made if the drainage of pleural effusion was <2 mL/kg/day/tube. Conventional anticoagulant therapy was started at night on the same day as the operation depending on coagulation function. Low-fat diet was administered in all patients during hospitalization. Renal replacement treatment was initiated when urine output dropped to <1 mL/kg/h and not responding to infusion of furosemide for 48 h. Other managements were based on the routine practice of Fuwai Hospital.

### Statistical Analysis

The R package “missForest” with random forest algorithm was used to impute the missing data. Differences in the distribution of data before and after imputation were evaluated. Student's *t*-test or the Mann-Whitney *U*-test, Chi-square test, or Fisher's exact test was used to compare data between yes and no persistent AKI, as appropriate. Logistic regression using backward elimination method was applied to identify the predictors for persistent AKI. The multivariate logistic model starts with the variables of *p* ≤ 0.2 in univariate logistic regression. Multicollinearity was considered before the multivariate logistic analysis. Univariate ordinary logistic regression was used to investigate the association between levels of persistent AKI (no-AKI, AKI-R, AKI-I/F) and in-hospital clinical outcomes. Other six subgroups were operationally picked out to examine whether the logistic model can be applied to other population of different characteristics. The calibration curve, Hosmer-Lemeshow goodness-of-fit test (HL test) and receiver operating characteristic curve (ROC) were used to evaluate the calibration and discrimination ability of the logistic model. *P* ≤ 0.05 was considered statistically significant. All analyzes were performed in R software (version 3.6.0).

## Results

### Characteristics of the Study Population

**Figure 1:**
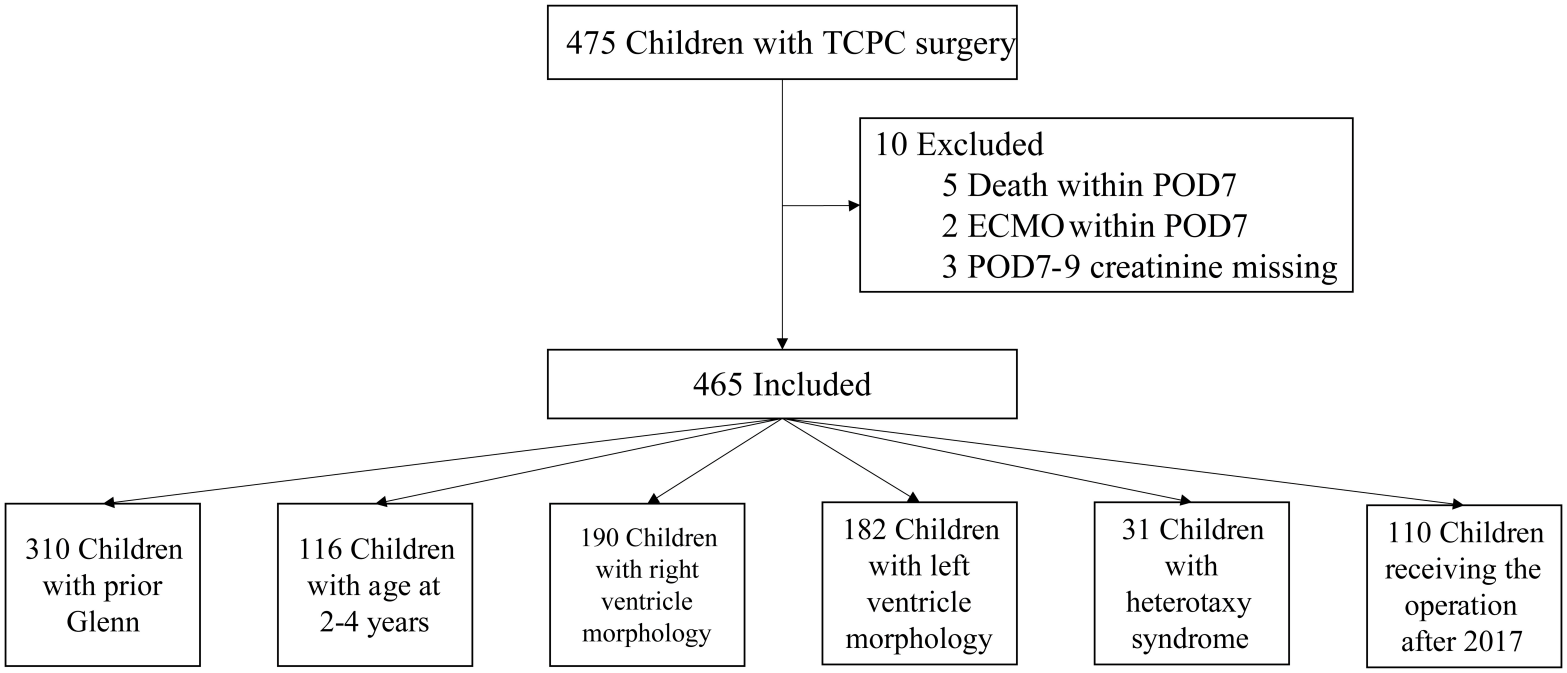
The flowchart of inclusion and exclusion criteria and the details of six subgroups.

From July 2010 to June 2019, a total of 475 children received TCPC surgery in our center. After the exclusion criteria, 465 cases were included in the final analysis (Figure 1). The rate of missing data in this study population ranged from 0.2 to 5.3% and 18 variables existed missing data. Complete data were seen in 380 (81.8%) cases. Among the six variables included in the final logistic model, two of them had missing data: POD0 maximal lactic acid with missing rate of 1.9% and POD0 total bilirubin with missing rate of 5.1%. The differences of distributions among data before and after imputation were not significant.

The median and interquartile range of the age and weight were 5.9(4.1, 9) years old and 18.4(15, 23.5) kg, respectively. The baseline eGFRs in no-AKI and AKI group were 102 ± 19.1 and 124 ± 36.5, respectively, and Five patients with eGFR <90 were all in no-AKI group. The top three diagnosis in these patients were unbalanced atrioventricular septal defect, tricuspid atresia and unbalanced double outlet right ventricle. Thirty-one patients (6.7%) had diagnosis of heterotaxy syndrome. Most patients (97%; *n* = 451) underwent extracardiac TCPC and had prior Glenn surgery (67%; *n* = 310). The percentages of right and left ventricle morphology were 40.9 and 39.1%, respectively. Moderate or more severe atrioventricular valve regurgitation was present in 13.5% of patients (*n* = 63). A percentage of 42.8% of patients (*n* = 199) had creation of fenestration. All patients underwent CPB and 30.3% (*n* = 141) patients were performed with aortic cross-clamping. The perioperative characteristics of the study patients with *p* < 0.2 in univariate analysis are displayed in Table 1 and the anatomy characteristics of the study patients are shown in Table 2.

**Table 1:** The perioperative characteristics of the study patients with *p* < 0.2 between yes and no persistent AKI.

**Variables**	**No-AKI (*n* = 393)**	**AKI (*n* = 72)**	***P*-value**	**Overall (*n* = 465)**
Prior B-T shunt surgery (yes)	15 (3.8%)	6 (8.3%)	0.11	21 (4.5%)
Heterotaxy syndrome (yes)	29 (7.4%)	2 (2.8%)	0.19	31 (6.7%)
Nakata index	206 ± 112	185 ± 88.8	0.103	203 ± 109
SpO2 before surgery, <80%	134 (34.1%)	35 (48.6%)	0.02	169 (36.3%)
Pre-operative pulmonary artery pressure (mmHg)	11.4 (9.0, 13.0)	11.0 (9.0, 13.0)	0.162	11.0 (9.0, 13.0)
Pre-operative platelet (×10^10^/L)	25.0 (20.1, 30.3)	23.0 (17.9, 28.8)	0.074	24.7 (19.9, 30.2)
Pre-operative albumin (g/L)	44.2 ± 3.6	43.5 ± 3.9	0.013	44.1 ± 3.7
Pre-operative lactate dehydrogenase (U/L)	272 (235, 318)	282 (245, 328)	0.171	272 (237, 319)
Pre-operative high-sensitivity C-reactive protein (mg/L)	0.53 (0.10, 1.51)	0.865 (0.23, 1.36)	0.168	0.57 (0.12, 1.48)
Norepinephrine use, n (%)	67 (17.0%)	17 (23.6%)	0.182	84 (18.1%)
Intraoperative red blood cells transfusion (mL/kg)	1.2 ± 4.5	2.2 ± 5.1	0.125	1.4 ± 4.6
Creation of fenestration (yes)	160 (40.7%)	39 (54.2%)	0.039	199 (42.8%)
Atrioventricular valve repair (yes)	44 (11.2%)	4 (5.6%)	0.199	48 (10.3%)
Intraoperative fluid balance (mL/kg)	7.5 ± 29.0	17.7 ± 28.9	0.015	9.1 ± 29.2
POD0 maximal lactic acid (mmol/L)	2.26 ± 1.46	2.76 ± 1.85	<0.001	2.34 ± 1.53
Post-operative pulmonary artery pressure (mmHg)	11.9 ± 2.7	12.8 ± 2.5	0.007	12.1 ± 2.7
RPP (mmHg)	49.4 ± 10.3	46.7 ± 7.9	0.013	49.0 ± 10.0
POD0 platelet (×10^10^/L)	19.1 (14.8, 24.3)	16.3 (12.1, 22.8)	0.02	18.9 (14.3, 24.0)
POD0 prothrombin time (seconds)	16.2 ± 2.0	17.8 ± 6.0	0.03	16.4 ± 3.1
POD0 aspartate aminotransferase (U/L)	54 (41, 78)	56 (45, 113)	0.152	54 (42, 79)
POD0 alkaline phosphatase (U/L)	103 (85, 121)	97 (78, 117)	0.071	102 (84, 120)
POD0 eGFR (mL/min/1.73 m^2^), <60	35 (8.9%)	16 (22.2%)	0.003	51 (11.0%)
POD0 TBIL (μmol/L), ≥38	201 (51.1%)	50 (69.4%)	0.006	251 (54.0%)
POD0 isoenzyme of creatine kinase-MB (U/L)	28(22,41)	32(22,47)	0.142	28(22,42)
POD0 lactate dehydrogenase (U/L)	411 (335, 518)	441 (349, 592)	0.105	413 (338, 528)

*AKI, persistent acute kidney injury; SpO2, peripheral oxygen saturation on room air before surgery; POD, post-operative day; RPP, renal perfusion pressure; eGFR, estimated glomerular filtration rate; TBIL, total bilirubin*.

**Table 2:** The demographic data of the study patients.

**Variables**	**No-AKI (*n* = 393)**	**AKI (*n* = 72)**	***P*-value**	**Overall (*n* = 465)**
Gender (male)	243 (61.8%)	45 (62.5%)	0.91	288 (61.9%)
Age (years)	5.8 (4.1, 9.0)	6.0 (4.0, 9.1)	0.76	5.9 (4.1, 9.0)
Weight (kg)	18.5 (15.5, 23.5)	18.0 (14.5, 24.1)	0.364	18.4 (15.0, 23.5)
Ventricle morphology *n* (%)			0.976	
Intermediated	79 (20.1%)	14 (19.4%)		93 (20.0%)
Left	153 (38.9%)	29 (40.3%)		182 (39.1%)
Right	161 (41.0%)	29 (40.3%)		190 (40.9%)
Original anatomy *n* (%)			0.977	
Unbalanced atrioventricular septal defect	112 (28.5%)	19 (26.4%)		131 (28.2%)
Tricuspid atresia	86 (21.9%)	14 (19.4%)		100 (21.5%)
Unbalanced double outlet right ventricle	73 (18.6%)	13 (18.1%)		86 (18.5%)
Double inlet ventricle	32 (8.1%)	7 (9.7%)		39 (8.4%)
Pulmonary atresia	33 (8.4%)	8 (11.1%)		41 (8.8%)
Complete transposition of great arteries	20 (5.1%)	4 (5.6%)		24 (5.2%)
Congenitally corrected transposition of the great arteries	27 (6.9%)	4 (5.6%)		31 (6.7%)
Others	10 (2.5%)	3 (4.2%)		13 (2.8%)
Priori glenn *n* (%)	266 (67.7%)	44 (61.1%)	0.286	310 (66.7%)
Systemic ventricle ejection fraction (<55%)	50 (12.7%)	6 (8.3%)	0.424	56 (12.0%)
Systemic ventricle end-diastolic diameter z-score	−0.67 (−4.58,2.94)	−0.62 (−4.31,2.25)	0.929	−0.67 (−4.49,2.80)

*AKI, persistent acute kidney injury*.

### Post-operative In-hospital Clinical Outcomes

One hundred sixty-four patients (35.3%) developed AKI between POD1 and POD3 after TCPC surgery and 72 patients (15.5%) had persistent AKI at POD7-9. Among the 164 patients with AKI occurring between POD1 and POD3, 3 patients had the stage of AKI-F and 66.7% (2/3) of them developed persistent AKI-F, 28 patients had the stage of AKI-I and 67.9% of them developed persistent AKI-I/R, and 133 patients had the stage of AKI-R and 38.4% patients developed persistent AKI-R. Among the 72 patients with persistent AKI, 2 patients had the stage of AKI-F, 13 patients had the stage of AKI-I and 57 patients had the stage of AKI-R.

The incidence of in-hospital mortality in pediatric patients following TCPC surgery was 1.7% (8/475). Development of persistent AKI after TCPC surgery was associated with: duration of mechanical ventilation (17 vs. 13 h); ICU stay (10 vs. 5 days); renal replacement treatment (30.6 vs. 6.9%); reintubation (11.1 vs. 2%); post-operative hospitalization costs (151 vs. 119 thousand RMB); and in-hospital mortality (2.8 vs. 0.3%). The impact of persistent AKI on in-hospital outcomes are presented in Table 3.

**Table 3:** Post-operative short-term outcomes in the study patients.

**Variables**	**AKI-I/F (*n* = 15)**	**AKI-R (*n* = 57)**	**No-AKI (*n* = 393)**	***P*-value**
Mechanical ventilation (hours)	80.0 (18.0, 224)	16.0 (9.00, 23.0)	13.0 (7.00, 21.0)	**0.001**
≥48 h	9 (60.0%)	11 (19.3%)	37 (9.4%)	** <0.001**
≥72 h	8 (53.3%)	11 (19.3%)	29 (7.4%)	** <0.001**
≥100 h	6 (40.0%)	9 (15.8%)	18 (4.6%)	** <0.001**
ICU stay (days)	14 (2,32)	3 (1,5)	3 (1,5)	** <0.001**
≥7 d	9 (60.0%)	10 (17.5%)	49 (12.5%)	**0.001**
≥14 d	7 (46.7%)	7 (12.3%)	22 (5.6%)	** <0.001**
Hospital stay (days)	33 (25,50)	18 (11,34)	20 (13,34)	0.266
≥21 d	12 (80.0%)	21 (36.8%)	174 (44.3%)	0.209
≥30 d	8 (53.3%)	19 (33.3%)	118 (30.0%)	0.091
≥45 d	5 (33.3%)	8 (14.0%)	44 (11.2%)	**0.026**
Pleural effusion (days)	13 (6,16)	6 (5,12)	9 (5,15)	0.54
≥7 d	9 (60.0%)	26 (45.6%)	248 (63.1%)	0.068
≥14 d	7 (46.7%)	12 (21.1%)	121 (30.8%)	0.967
≥21 d	2 (13.3%)	6 (10.5%)	64 (16.3%)	0.34
Renal replacement treatment *n* (%)	8 (53.3%)	14 (24.6%)	27 (6.9%)	** <0.001**
Reintubation *n* (%)	4 (26.7%)	4 (7.0%)	8 (2.0%)	** <0.001**
Vasopressin use *n* (%)	9 (60.0%)	7 (12.3%)	88 (22.4%)	0.809
Mortality *n* (%)	1 (6.7%)	1 (1.8%)	1 (0.3%)	**0.028**
Post-operative costs (thousand RMB)	126 (81.9, 280)	98.5 (67.2, 140)	100 (77.7, 135)	**0.008**
Total costs (thousand RMB)	132 (89.4, 284)	101 (71.6, 144)	104 (82.9, 141)	**0.01**

*AKI, persistent acute kidney injury; I/F, injury/failure; R, risk; ICU, intensive care unit*.

### Prediction of Persistent AKI Using Logistic Regression Model

Among the 73 clinical variables, 26 variables with *p* ≤ 0.2 in univariate analysis were put into the multivariate logistic analysis, and SpO2 upon admission the hospital, intraoperative fluid balance, POD0 maximal lactic acid, RPP, POD0 estimated glomerular filtration rate and POD0 total bilirubin were independent predictors for persistent AKI. The odd ratios of the logistic analysis are shown in Table 4.

**Table 4:** Univariate and multivariate logistic analysis odds ratios for persistent acute kidney injury.

**Variables**	**Univariate analysis**		**Multivariate analysis**	
	**OR (95%CI)**	***p***	**OR (95%CI)**	***p***
Prior B-T shunt surgery (yes vs. no)	2.29 (0.86, 6.12)	0.119		
Heterotaxy syndrome (yes vs. no)	0.36 (0.08, 1.54)	0.112		
Nakata index	0.998 (0.995, 1.001)	0.123		
SpO2 before surgery (%), <80 vs. ≥80	**1.82 (1.1, 3.03)**	**0.02**	**1.72 (1.01, 2.94)**	**0.045**
Pre-operative pulmonary artery pressure (mmHg)	0.94 (0.87, 1.02)	0.146		
Pre-operative platelet (×10^10^/L)	0.97 (0.94, 1)	0.084		
Pre-operative albumin (g/L)	0.95 (0.89, 1.02)	0.154		
Pre-operative alamine aminotransferase (U/L)	1.02 (0.99, 1.05)	0.147		
Pre-operative lactate dehydrogenase (U/L)	1.003 (0.999, 1.006)	0.141		
Pre-operative high-sensitivity C-reactive protein (mg/L)	0.92 (0.8, 1.04)	0.149		
Norepinephrine use, *n* (%)	1.5 (0.82, 2.75)	0.196		
Intraoperative red blood cells transfusion (mL/kg)	1.04 (0.99, 1.09)	0.119		
Creation of fenestration (yes vs. no)	1.72 (1.04, 2.85)	0.035		
Atrioventricular valve repair (yes vs. no)	0.47 (0.16, 1.34)	0.121		
Intraoperative fluid balance (mL/kg)	**1.01 (1.002, 1.02)**	**0.005**	**1.01 (1.001, 1.02)**	**0.038**
POD0 maximal lactic acid (mmol/L)	**1.19 (1.03, 1.37)**	**0.019**	**1.18 (1.01, 1.36)**	**0.034**
Post-operative pulmonary artery pressure (mmHg)	1.13 (1.03, 1.24)	0.01		
RPP (mmHg)	**0.97 (0.94, 0.99)**	**0.03**	**0.97 (0.94, 0.99)**	**0.044**
POD0 platelet (×10^10^/L)	0.96 (0.93, 0.99)	0.016		
POD0 prothrombin time (seconds)	1.14 (1.05, 1.24)	<0.001		
POD0 aspartate aminotransferase (U/L)	1.001 (1.0001, 1.0013)	0.049		
POD0 alkaline phosphatase (U/L)	0.99 (0.98, 1.002)	0.096		
POD0 eGFR (mL/min/1.73 m^2^), ≥60 vs. <60	**0.34 (0.18, 0.66)**	**0.002**	**0.46 (0.23, 0.93)**	**0.03**
POD0 TBIL (μmol/L), ≥38 vs. <38	**2.17 (1.27, 3.72)**	**0.004**	**1.85 (1.05, 3.26)**	**0.032**
POD0 isoenzyme of creatine kinase-MB (U/L)	1.008 (0.999, 1.02)	0.107		
POD0 lactate dehydrogenase (U/L)	1.001 (1.0002, 1.001)	0.002		

*SpO2, peripheral oxygen saturation on room air before surgery; POD, post-operative day; RPP, renal perfusion pressure; eGFR, estimated glomerular filtration rate; TBIL, total bilirubin*.

### Performance of the AKI Model in the Total Cohort and Different Subgroups

**Figure 2:**
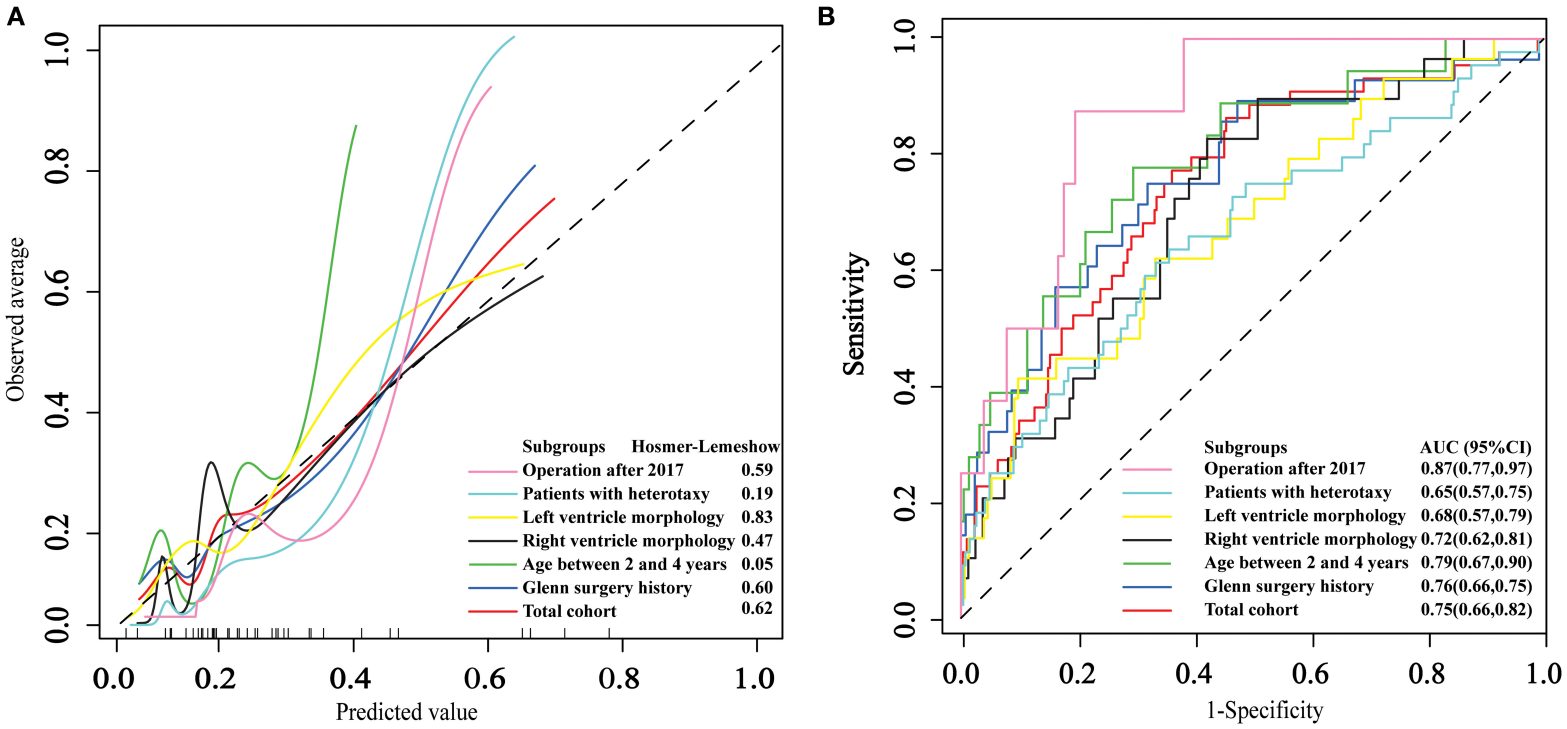
The application and performance of the logistic model in the total cohort and six subgroups. **(A)** Calibration curves of the logistic model. **(B)** Receiver operating characteristic curves for persistent acute kidney injury.

The area under ROC curve (AUC) and *p*-value of HL test in the total cohort was 0.75(0.66, 0.82) and 0.62, respectively. The strongest discrimination ability was achieved in subgroup patients who underwent the surgery after 2017 with an AUC of 0.87(0.77, 0.97). The moderate discrimination ability (AUC ≥ 0.7) were also achieved in other 3 subgroups, which included patients with prior Glenn surgery, patients between 2 and 4 years old and patients diagnosed with right ventricle morphology. However, poor discrimination (AUC <0.7) were achieved in subgroup patients with heterotaxy syndrome and subgroup patients diagnosed with left ventricle morphology. Good fitness (HL test *p* ≥ 0.05) were found in all six subgroups. The calibration and ROC curves are shown in Figure 2.

## Discussions

This study demonstrated that the development of AKI between POD1 and POD3 was common (35.3%, 164/465), and nearly half of them (72/164) had persistent AKI later (15.5% of total cohort). Patients with persistent AKI were strongly associated with poor in-hospital outcomes, including prolonged mechanical ventilation, prolonged ICU stay, higher rates of reintubation and renal replacement treatment, higher hospitalization costs and in-hospital mortality. The independent predictors for persistent AKI included SpO2 at admission, intraoperative fluid balance, POD0 maximal lactic acid, RPP, POD0 moderate-to-severe kidney injury and POD0 total bilirubin. The logistic model including these predictors fitted well among total cohort and subgroups. Our findings are helpful to identify post-operative high-risk patients, and early adoption of strategies including avoiding hypotension, using inotropic medication and creation of fenestration if necessary, early extubation, etc. to prevent hypoperfusion and the increase of pulmonary vascular resistance, might reduce the occurrence of persistent AKI.

In this study, we chose the pRIFLE criteria for the diagnosis of AKI because the pRIFLE criteria detected AKIs in pediatric cardiac patients more sensitively than other methods (15). The incidence of persistent AKI might be underestimated because we didn't combining the urine output to define AKI. The incidence of AKI varies under different criteria of AKI, most of which are based on the maximal creatinine level between POD1 and POD3. In our study, the incidence of AKI occurring between POD1 and POD3 was similar to previous reports (35.3 vs. 42% or 39.9%) (9, 10). Among these patients, nearly half of them (72/164) developed persistent AKI and required more medical attentions, such as mechanical ventilation support, renal replacement treatment and longer ICU stay. However, the longer follow-up time of post-operative serum creatinine is needed in future studies, although the definition met the criteria in the previous studies (13, 16).

In previous studies (9, 10), renal perfusion is critical to the development of AKI after cardiac surgery. We found that the indicator of post-operative RPP was an independent predictor for persistent AKI in our study. Compared to patients without persistent AKI, an increase in central venous pressure and a decline of mean arterial pressure were observed in those with persistent AKI, particularly poorer pressure changes in patients with severe persistent AKI (AKI-I/F). Higher levels of POD0 maximal lactic acid, which indicates a state of hypoperfusion, was also observed in patients with persistent AKI. Elevated central venous pressure and decreased systemic blood pressure after surgery in these patients indicate decreased renal perfusion. On the one hand, increased central venous pressure can be induced by positive mechanical ventilation, as well as increased pulmonary vascular resistance related to lung injury and post-operative fluid over-loading; On the other hand, low systemic vascular resistance related to CPB and inflammatory response, low cardiac output after the surgery, diuretic use, hepatic dysfunction and blood loss might result in decreased systemic blood pressure. Thus, strategies that reduce the negative impacts of these perioperative events might improve renal perfusion, although the efficacy of these strategies should be evaluated in future studies.

In our study, the three strongest independent predictors for persistent AKI were low pre-operative SpO2 upon admission, POD0 moderate-to-severe kidney injury and increased POD0 total bilirubin. Patients with low pre-operative SpO2 might be exposed to more chronic hypoxic injuries, which poses the kidney at higher risk of injuries related to CPB after TCPC surgery (17), and Patients with low pre-operative SpO2 might also suffer from the poor development of pulmonary arteries, complex anatomy malformation and collateral circulation, which might increase the risk of AKI. Increased POD0 total bilirubin suggested more severe hemolysis (18) and hepatic injuries after TCPC surgery, which may result in post-operative AKI. Not surprisingly, POD0 moderate-to-severe kidney injury was a strong independent risk factor for persistent AKI later.

Some of the identified risk factors for persistent AKI are not modifiable like pre-operative SpO2, POD0 eGFR, and POD0 total bilirubin, but they can be utilized to stratify patients with high risk for persistent AKI after TCPC surgery and other factors like fluid balance, POD0 maximal lactic acid and RPP, may give us some hints that an early strategy to maintain a more exquisite management of fluid and vasoactive inotropic drugs administration, and to avoid the increase of pulmonary vascular resistance to improve renal perfusion could reduce the occurrence of persistent AKI in these high-risk patients.

The population in our study was different from that in previous studies (9, 10). Firstly, patients in our study were generally older at presentation for the surgery (median age, 5.9 vs. 2.7 or 3.8). This is a special issue in China, because the high rates of patients with low socioeconomic status in China may create barriers to timely surgical care for these patients (19). Secondly, the predominant diagnosis in our study was unbalanced atrioventricular septal defect, while the predominant diagnosis in previous studies was hypoplastic left heart syndrome. Therefore, the percentage of intermediated morphology in our study was up to 20%. Thirdly, the proportion of patients (67%) with prior history of Glenn procedure, especially those undergoing TCPC surgery in early years, was lower than those in developed countries. This might be accounted by the fact that some parents in China may not afford their children to receive a second surgery after Glenn procedure because of higher financial burdens. Additionally, some surgeons in China did not fully appreciate the clinical benefits of Glenn procedure in relatively older patients until recent years, and the proportion of patients with prior Glenn procedure rose to 82% until 2017. Despite these differences, subgroup analysis in our study indicates that our model fitted well among the total cohort and all six subgroups. Therefore, the model derived from our study had clinical values not only for the Chinese population, but also for patients of different characteristics from other countries.

## Limitations

There are several limitations in this study. Firstly, the limitation of this retrospective study was similar to those in other retrospective studies, which included certain unknown heterogeneities, no causal conclusions, patient selection bias, missing data, etc., thus future prospective cohort studies or RCTs still need to explore the clear relations between potential factors and persistent AKI. Secondly, because some variables can't be achieved routinely, some potential variables were not included in our study, such as pre-operative cardiac catheterization parameters, cardiac troponin I level, use of dialysis and anti-inflammatory medications, partial pressure of oxygen (PaO2), etc., the impacts of these factors still need further study. Thirdly, moderate discrimination power of the model (AUC = 0.75) in the total cohort was achieved, which may be due to the heterogeneity of the population and the large-range operation years. Fourthly, the lack of long-term follow-up of renal function and other clinical outcomes limit our study. Fifthly, there are many criteria for AKI diagnosis after cardiac surgery based on serum creatinine or urine output, such as pRIFLE, kidney disease improving global outcomes (KDIGO) criteria, acute kidney injury network (AKIN) criteria and so on. Different criteria might result in different incidence and risk factors. Therefore, the incidence and risk factors of AKI based on KDIGO or AKIN in patients with TCPC surgery still need future studies. Finally, the risk factors of AKI in excluded patients (with death or extracorporeal membrane oxygenation) should also be further studied in future.

## Conclusions

AKI occurring between POD1 and POD3 was common (35.3%) and the incidence of persistent AKI was 15.5%. Patients with persistent AKI required more medical attentions, including mechanical ventilation support, renal replacement treatment and longer ICU stay, and were more likely to have worse in-hospital outcomes, including reintubation, higher hospitalization costs and in-hospital mortality. Six variables, including SpO2 at admission, intraoperative fluid balance, POD0 maximal lactic acid, RPP, POD0 moderate-to-severe kidney injury, and POD0 total bilirubin, were identified as independent predictors for persistent AKI. Our model fitted well in the total cohort as well as in subgroup analysis, but further external validation is warranted before its use in clinical practice.

## Data Availability Statement

The datasets generated for this study will not be made publicly available because of data protection policy in our hospital.

## Ethics Statement

The studies involving human participants were reviewed and approved by Research Ethics Board of Fuwai Hospital. Written informed consent for participation was not provided by the participants' legal guardians/next of kin because: Informed consents were waived because this study was a retrospective study.

## Author Contributions

FY, SY, and YJ proposed the idea of this investigation. HW, YL, and XW were responsible for the collection of data and material. QL helped with the statistical analysis and wrote the manuscript. FY, SY, YJ, ZS, and CX helped to revise the manuscript. All authors contributed extensively to the work presented in this paper, read, and approved the final manuscript.

## Conflict of Interest

The authors declare that the research was conducted in the absence of any commercial or financial relationships that could be construed as a potential conflict of interest.
